# Biochar-Coconut Shell Mixtures as Substrates for *Phalaenopsis* ‘Big Chili’

**DOI:** 10.3390/plants14142092

**Published:** 2025-07-08

**Authors:** Yun Pan, Daoyuan Chen, Yan Deng, Shunshun Wang, Feng Chen, Fei Wang, Luyu Xue, Yanru Duan, Yunxiao Guan, Jinliao Chen, Xiaotong Ji, Donghui Peng

**Affiliations:** Key Laboratory of National Forestry and Grassland Administration for Orchid Conservation and Utilization at College of Landscape Architecture and Art, The Innovation and Application Engineering Technology Research Center of Ornamental Plant Germplasm Resources in Fujian Province, National Long Term Scientific Research Base for Fujian Orchid Conservation, Fujian Agriculture and Forestry University, Fuzhou 350002, Chinachendaoyuan0527@163.com (D.C.); yandeng@fafu.edu.cn (Y.D.); shunshunwang@fafu.edu.cn (S.W.); fengchen@fafu.edu.cn (F.C.); xueluyu2023@163.com (L.X.); dyr239921@163.com (Y.D.); guanyunxiao@fafu.edu.cn (Y.G.);

**Keywords:** *Phalaenopsis* ‘Big Chili’, biochar, plant growing medium, plant growth

## Abstract

*Phalaenopsis* is a widely cultivated ornamental plant of considerable economic value worldwide. However, traditional growing medium, sphagnum moss, is limited and non-renewable. It also decomposes slowly and is prone to environmental issues. Therefore, there is an urgent need to identify more environmentally friendly and efficient alternatives. Biochar, a sustainable material with excellent physical and chemical properties, has been recognized as an effective promoter of plant growth. In this study, we investigated the influence of biochar derived from three raw materials (corn straw, bamboo, and walnut) mixed1 with coconut shell at ratios of 1:2, 1:10, and 4:1, on the growth of *Phalaenopsis* ‘Big Chili’. Over a 150-day controlled experiment, we evaluated multiple growth parameters, including plant height, crown width, total root length, total projected area, total surface area, and root volume. Compared to the traditional growing medium, the optimal biochar-coconut shell mixture (maize straw biochar: coconut shell = 1:2) increased plant height and crown width by 7.55% and 6.68%, respectively. Root metrics improved substantially, with total root length increasing by 10.96%, total projected area by 22.82%, total surface area by 22.14%, and root volume by 38.49%. Root biomass in the optimal treatment group increased by 42.47%, while aboveground and belowground dry weights increased by 6.16% and 77.11%, respectively. These improvements were closely associated with favorable substrate characteristics, including low bulk density, high total and water-holding porosity, moderate aeration, and adequate nutrient availability. These findings demonstrate that substrate characteristics critically influence plant performance and that biochar–coconut shell mixtures, particularly at a 1:2 ratio, represent a viable and sustainable alternative to sphagnum moss for commercial cultivation of *Phalaenopsis*.

## 1. Introduction

*Phalaenopsis* Blume, with its unique flower shape, bright colors, and long flowering period, is one of the most significant orchid species in the global flower industry, particularly in markets for cut flowers and potted plants [1,2]. With the increasing demand for high-value ornamental plants in both domestic and international markets, *Phalaenopsis* has gradually emerged as a key economic crop [3]. However, it has very strict requirements for its cultivation environment, particularly the selection and management of the substrate, making the choice of growing medium a crucial factor in the production of *Phalaenopsis*.

Sphagnum moss is a traditional substrate widely used in *Phalaenopsis* cultivation due to its excellent water retention, good aeration, and moderate nutrient content [4,5]. However, the long-term use of sphagnum moss raises environmental and practical concerns [6]. Harvesting primarily depends on wetlands and peatlands, and excessive harvesting can not only damage wetland ecosystems but also lead to species extinction, further exacerbating ecological disruption [7]. Moreover, sphagnum moss grows slowly and has a long recovery period after harvesting [8]. It is also costly to collect and is prone to waterlogging during the later stages of cultivation, which can lead to root rot in *Phalaenopsis* and hinder its normal growth [9]. Consequently, the sustainable use of sphagnum moss is increasingly challenged, highlighting the urgent need for more environmentally friendly and sustainable alternatives.

Biochar, a carbon-rich material produced through the high-temperature pyrolysis of waste biomass, has been widely applied in soil remediation, carbon sequestration, agricultural production, and other fields due to its favorable physical and chemical properties [10]. Studies have shown that biochar can significantly enhance soil quality by improving its physicochemical properties. As an effective adsorbent, biochar improves soil fertility, increases water retention, and promotes plant growth, development, and stress resistance [11,12]. The performance of biochar varies depending on factors such as pyrolysis temperature, feedstock type, and microstructure, resulting in different effects on plant growth [13]. For instance, bamboo biochar has been shown to substantially increase crop yields in agriculture [14], while the addition of an appropriate proportion of straw biochar has promoted root development and biomass accumulation in the cultivation of *Ardisia crenata* [15]. Moreover, biochar can improve the efficient utilization of nutrients, such as nitrogen, phosphorus, and potassium, by enhancing nutrient retention and reducing nutrient loss, further supporting plant growth [16]. Additionally, its synergistic effects with plant growth-promoting microorganisms have been found to boost disease resistance and improve tomato productivity [17]. Beyond its agricultural applications, biochar plays a crucial role in environmental protection by reducing greenhouse gas emissions [18]. With its low-cost production, abundant raw material sources, and wide range of benefits, biochar is increasingly viewed as a promising tool for sustainable development.

Coconut shell is an organic material derived from the shell or mesocarp of the coconut fruit. Due to its renewable nature, it offers significant environmental benefits [19]. Coconut shells have excellent air permeability and water retention properties, and their low cost makes them a widely used substrate in soilless cultivation [20]. It effectively promotes root development, prevents water accumulation, and enhances plant growth and health, making it a popular choice for modern cultivation [21]. However, studies have indicated that coconut shell substrates alone may have limitations, including insufficient nutrient supply and slow plant growth. To address these issues, composite substrates incorporating coconut shells with other materials are gaining popularity. Consequently, coconut shells are increasingly used as components in composite substrates to improve overall performance and better support plant growth. Previous studies have demonstrated that a biochar-coconut shell mixture, when combined in an appropriate ratio, yields positive results in promoting plant growth [22]. Such a mixture has been shown to enhance substrate performance and foster more effective plant growth [23,24]. However, the potential of these mixed substrates, particularly for *Phalaenopsis* cultivation, remains underexplored.

The aim of this study was to investigate the effects of different biochar–coconut shell mixtures on the growth, physiological characteristics, and photosynthetic pigment content of *Phalaenopsis* ‘Big Chili’. The physicochemical properties of the substrates were systematically determined, and plant performance was comprehensively analyzed. This study evaluated the potential of these mixtures as sustainable alternatives to traditional peat moss substrates and provided a theoretical basis for selecting improved substrates for *Phalaenopsis* cultivation.

## 2. Results

### 2.1. Physical and Chemical Properties of the Substrate

**Figure 1:**
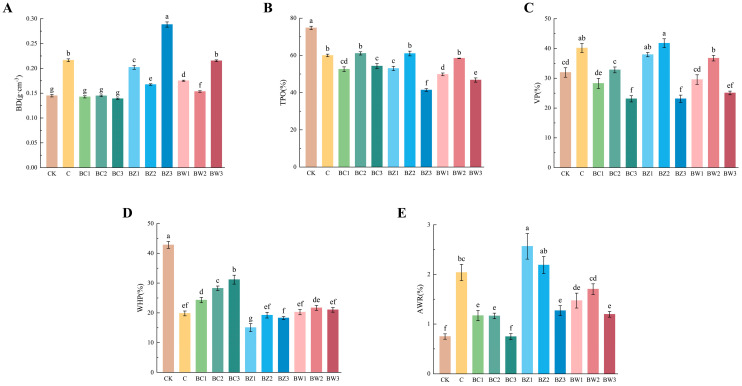
Physical properties of the treatment groups. (**A**) bulk density (BD), (**B**) total porosity (TPO), (**C**) ventilation porosity (VP), (**D**) water-holding porosity (WHP), and (**E**) air-to-water ratio (AWR). Different letters indicate significant differences among treatments based on Duncan’s multiple range test (*p* ≤ 0.05). Error bars represent the standard deviation (*n* = 3).

The physical properties of the substrate varied significantly among the treatments (Figure 1, Table S1). Bulk density (BD) ranged from 0.139 to 0.288 g·cm^−3^, with BZ3 nearly doubling BC3 (ANOVA: *F*_10,22_ = 310.585, *p* < 0.001). BD generally increased with BZ and BW content but decreased with BC addition. The total porosity (TPO) ranged from 41.41% to 61.00% (*F*_10,22_ = 79.834, *p* < 0.001), showing a downward trend with higher BZ and BW contents, while BC treatments exhibited a slight increase. Notably, the TPO of the 1:10 biochar mixture closely resembled that of pure coconut shell biochar (C), although it was still lower than that of CK. Ventilation porosity (VP) decreased with increasing biochar content across all treatments (*F*_10,22_ = 27.608, *p* < 0.001), while water-holding porosity (WHP) was highest in CK and significantly reduced in other treatments (*F*_10,22_ = 62.395, *p* < 0.001), although it slightly increased in BC at higher ratios. The air-to-water ratio (AWR) also varied significantly, with BZ1 and BZ2 showing the highest values (*F*_10,22_ = 19.828, *p* < 0.001).

**Figure 2:**
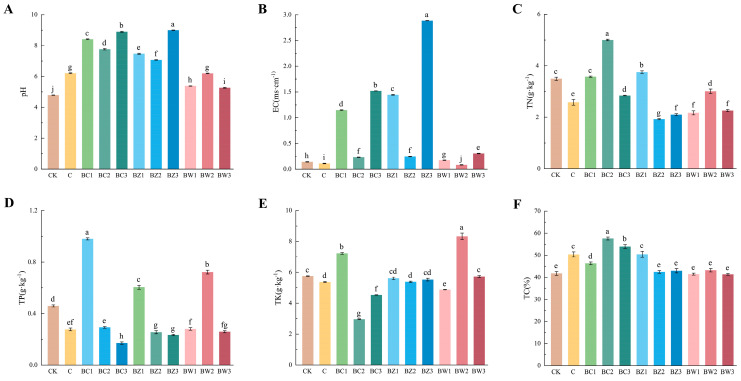
Chemical properties of the treatment groups. (**A**) pH, (**B**) electrical conductivity (EC), (**C**) total nitrogen (TN), (**D**) total phosphorus (TP), (**E**) total potassium (TK), (**F**) total carbon (TC). Different letters indicate significant differences among treatments based on Duncan’s multiple range test (*p* ≤ 0.05). Error bars represent the standard deviation (*n* = 3).

The chemical properties also differed significantly among the treatments (Figure 2 and Table S2). The pH ranged from 4.80 (CK) to 9.00 (BZ3), showing alkaline tendencies in the BC and BZ treatments, while BW and C maintained a slightly acidic to neutral pH (5.26–6.21) (*F*_10,22_ = 3122.443, *p* < 0.001). The electrical conductivity (EC) ranged from 0.085 to 2.885 mS·cm^−1^, all within suitable levels for orchid cultivation (*F*_10,22_ = 31,170.907, *p* < 0.001). Total nitrogen (TN) reached 5.00 g·kg^−1^ in BC2, nearly twice that of CK (*F*_10,22_ = 253.858; *p* < 0.001). The total phosphorus (TP) ranged from 0.171 to 0.980 g·kg^−1^, with BC1 achieving the highest value (*F*_10,22_ = 608.869, *p* < 0.001), while the total potassium (TK) peaked in BW2 at 8.33 g·kg^−1^ (*F*_10,22_ = 295.172, *p* < 0.001). Total carbon (TC) peaked in BC2 (57.57%) (*F*_10,22_ = 46.349; *p* < 0.001). These results demonstrate that both the type and proportion of biochar significantly influenced the nutrient composition and pH of the substrate, with each biochar type contributing distinct chemical characteristics.

### 2.2. Morphological Traits of the Aboveground Part of Phalaenopsis

**Figure 3:**
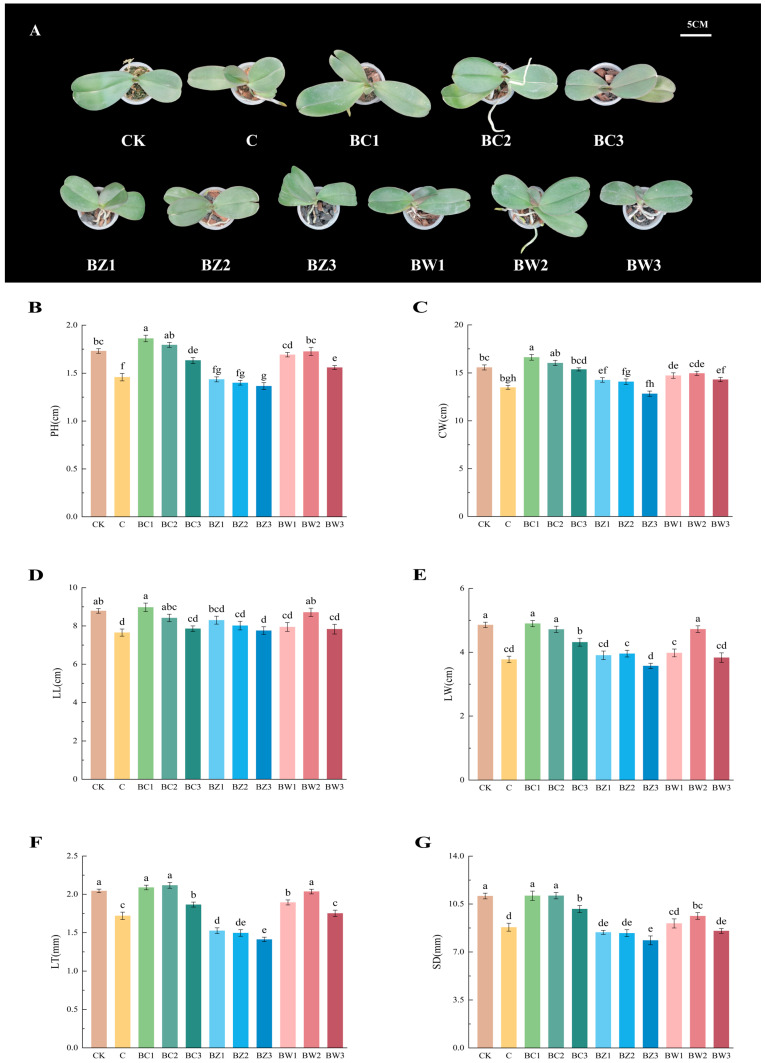
Aboveground growth traits of *Phalaenopsis* ‘Big Chili’ under different substrate treatments for 150 days. Effects of different treatments on the aboveground phenotypes of *Phalaenopsis*. (**A**) Representative plant images under each treatment (CK, C, BC1, BC2, BC3, BZ1, BZ2, BZ3, BW1, BW2, and BW3), photographed in November 2024 (left to right). (**B**) plant height (PH), (**C**) crown width (CW), (**D**) leaf length (LL), (**E**) leaf width (LW), (**F**) leaf thickness (LT), and (**G**) stem diameter (SD). Different colors represent different months. Values are expressed as mean ± SE (*n* = 15). Different letters indicate significant differences at *p* ≤ 0.05 (Duncan’s test).

The aboveground growth rate of *Phalaenopsis* ‘Big Chili’ varied among the substrate treatments (Figure 3A–G). BC1 and BC2 showed significantly greater plant height (*F*_10,154_ = 31.530, *p* < 0.001) and crown width (*F*_10,154_ = 19.743, *p* < 0.001) than CK, while BC1, BC2, and BW2 exhibited comparable leaf length (*F*_10,154_ = 5.125, *p* < 0.001), leaf width (*F*_10,154_ = 19.258, *p* < 0.001), leaf thickness (*F*_10,154_ = 49.773, *p* < 0.001), and stem diameter (*F*_10,154_ = 21.693, *p* < 0.001) to CK. In contrast, BZ treatments generally showed poor performance at this stage.

To better understand these differences, dynamic changes from 30 to 120 days were assessed (Figure S1). At 30 days, most treatments showed minimal differences, except that the crown width and leaf size in BZ substrates were already significantly lower. From 60 to 120 days, growth rates diverged more clearly, with BC treatments showing the fastest growth, BW showing moderate growth, and BZ showing consistently the slowest growth.

### 2.3. Morphological Traits of the Belowground Part of Phalaenopsis ‘Big Chili’

**Figure 4:**
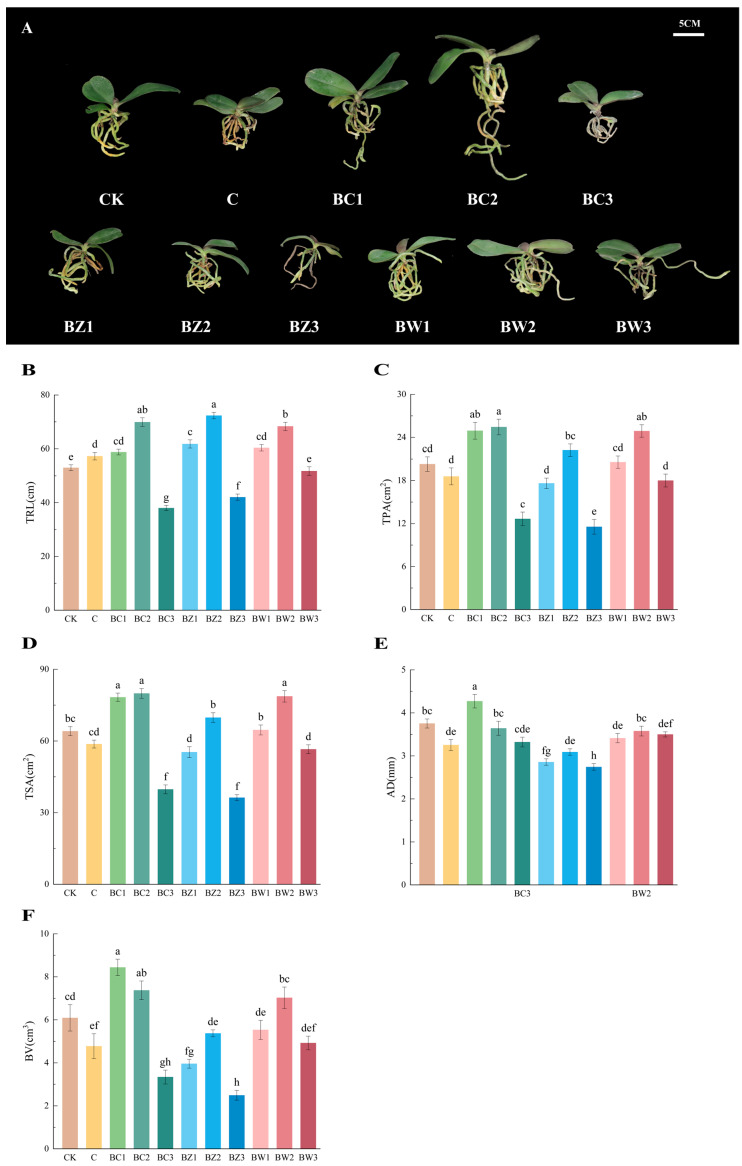
Effects of different treatments on root traits of *Phalaenopsis*. (**A**) Representative plant images under each treatment (CK, C, BC1, BC2, BC3, BZ1, BZ2, BZ3, BW1, BW2, and BW3), photographed in November 2024 (left to right). (**B**) Total root length, (**C**) total projected area, (**D**) total surface area, (**E**) average diameter, and (**F**) root volume. Values are expressed as mean ± SE (*n* = 6). Different letters indicate significant differences at *p* ≤ 0.05 (Duncan’s test).

BC1 and BC2 significantly outperformed CK in terms of total root length (TRL), total projected area (TPA), total surface area (TSA), average diameter (AD), and root volume (RV). Among all treatments, BC2 showed the highest TRL, TPA, AD, and RV (Figure 4A–F; TRL: *F*_10,55_ = 68.115, *p* < 0.001; TPA: *F*_10,55_ = 22.907, *p* < 0.001; TSA: *F*_10,55_= 57.785, *p* < 0.001; AD: *F*_10,55_ = 14.739, *p* < 0.001; RV: *F*_10,55_= 19.188, *p* < 0.001).

BW2 also exhibited strong performances in TRL, TPA, and TSA. In contrast, root growth in BC3, BZ3, and BW3 was markedly reduced, likely due to the inhibitory effects of excessive biochar.

### 2.4. Biomass Parameters of Phalaenopsis ‘Big Chili’

**Figure 5:**
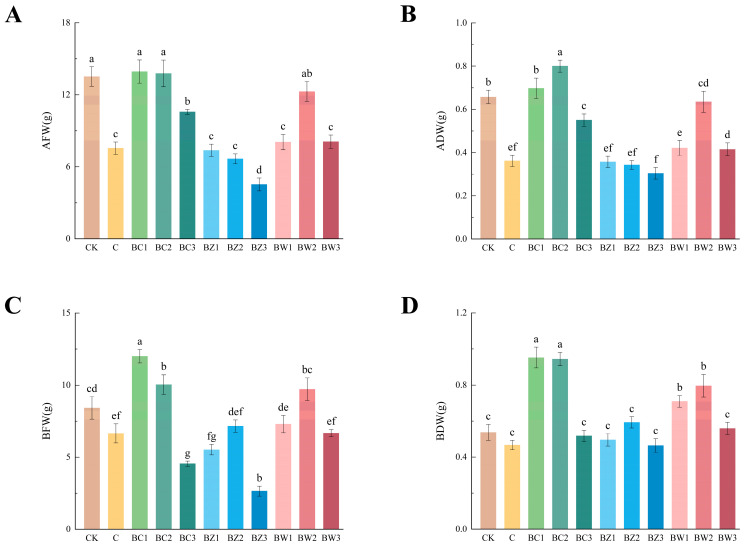
Effects of different treatments on the biomass of *Phalaenopsis*. (**A**,**B**) Aboveground fresh and dry weights; (**C**,**D**) belowground fresh and dry weights. Values are expressed as mean ± SE (*n* = 6). Different letters indicate significant differences at *p* ≤ 0.05 (Duncan’s test).

In the aboveground portion, the fresh weights of BC1, BC2, BW2, and CK were not significantly different. However, BW2 showed significantly higher dry weight than CK, indicating improved shoot biomass accumulation (Figure 5A,B; AFW: *F*_10,55_ = 22.358, ADW: *F*_10,55_ = 27.706, both *p* < 0.001). In the belowground portion, BC1 and BC2 produced significantly greater fresh and dry weights than CK, with BC2 showing the highest root biomass (Figure 5C,D; BFW: *F*_10,55_ = 23.614, BDW: *F*_10,55_ = 20.463, both *p* < 0.001).

### 2.5. Phalaenopsis ‘Big Chili’ Physiological Parameters

**Figure 6:**
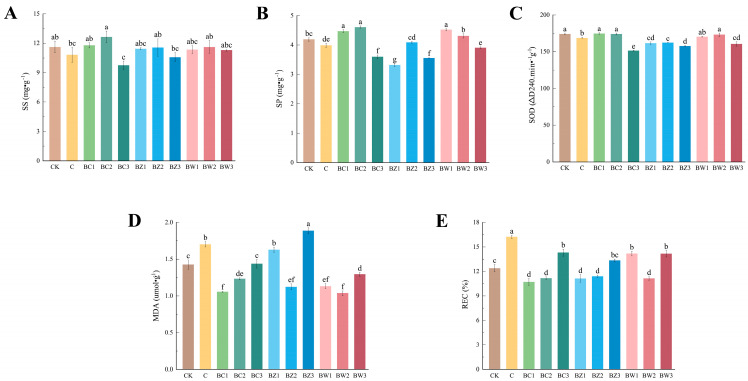
Effects of different treatments on the physiological parameters of *Phalaenopsis*. (**A**–**E**) (**A**) Soluble sugar, (**B**) soluble protein, (**C**) superoxide dismutase, (**D**) malondialdehyde, and (**E**) relative electrical conductivity. Values are expressed as mean ± SE (*n* = 3). Different letters indicate significant differences at *p* ≤ 0.05 (Duncan’s test).

The soluble sugar (SS) and soluble protein (SP) contents varied among the treatments (Figure 6A,B). SS did not differ significantly across treatments (*F*_10,22_ = 1.925, *p* = 0.096), although relatively higher values were observed in BC1 and BC2, and lower values in BC3 and BZ3. SP showed highly significant differences (*F*_10,22_ = 69.800, *p* < 0.001), with the highest values recorded for BC1, BC2, and BW1. Antioxidant and stress-related indicators also showed significant treatment effects. Superoxide dismutase (SOD) activity varied significantly (*F*_10,22_ = 31.164, *p* < 0.001), with reduced activity observed in the high-ratio biochar treatments (Figure 6C). Malondialdehyde (MDA) content and relative electrical conductivity (REC) also differed significantly among treatments (MDA: *F*_10,22_ = 54.311, *p* < 0.001; REC: *F*_10,22_ = 31.038, *p* < 0.001), with both increasing in high-ratio groups (Figure 6D,E).

### 2.6. Phalaenopsis ‘Big Chili’ Photosynthetic Pigments

**Figure 7:**
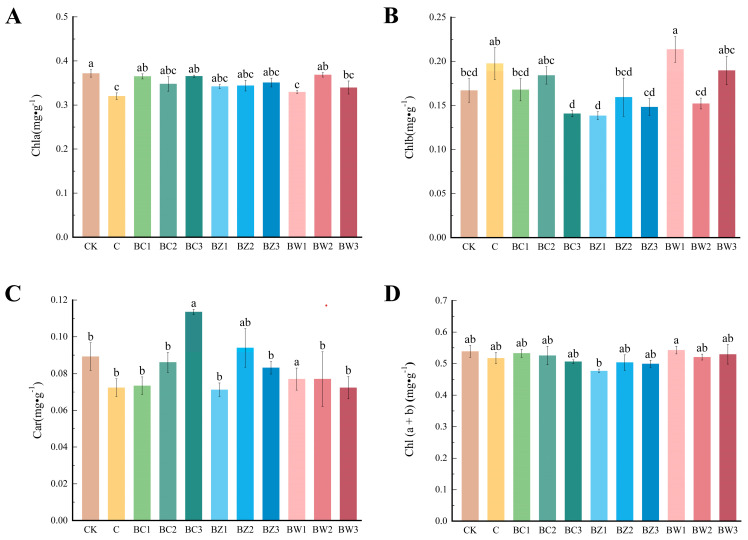
Effects of different treatments on chlorophyll and carotenoid contents in *Phalaenopsis*. (**A**–**D**) (**A**) Chlorophyll a, (**B**) Chlorophyll b, (**C**) carotenoids, (**D**) total chlorophyll. Values are expressed as mean ± SE (*n* = 3). Different letters indicate significant differences at *p* ≤ 0.05 (Duncan’s test).

Chlorophyll a, the primary photosynthetic pigment, was significantly lower in the C (*F*_10,22_ = 3.214, *p* = 0.011) and BW1 (*F*_10,22_ = 3.544, *p* = 0.006) groups, with the lowest values observed in the C and BW1 groups (Figure 7A). In contrast, chlorophyll b levels were relatively high in these treatments (Figure 7B). The carotenoid content was highest in BZ1 (*F*_10,22_ = 3.206, *p* = 0.011), whereas the chlorophyll b content was lowest (Figure 7C). The total chlorophyll (a + b) content showed no significant differences among the treatments (*F*_10,22_ = 1.160, *p* = 0.366), indicating that the substrate had a limited effect on pigment synthesis (Figure 7D).

### 2.7. Relationship Between the Physicochemical Properties of Substrate and the Characteristics of Phalaenopsis

The physical properties of the substrate, including bulk density (BD) and air–water ratio (AWR), showed significant negative correlations with *Phalaenopsis* ‘Big Chili’ growth traits (e.g., BD vs. stem diameter (SD), r = −0.797, *p* = 0.003; whereas total porosity (TPO) and water-holding porosity (WHP) were positively correlated. Notably, ventilation porosity (VP) was negatively correlated with aboveground traits but positively correlated with root-related traits (e.g., VP vs. total root length (TRL), r = 0.781, *p* = 0.005).

Among the chemical properties, pH and EC were not significantly correlated with plant growth. In contrast, total nitrogen (TN), phosphorus (TP), potassium (TK), and carbon (TC) were positively correlated with both shoot and root traits, with TN and TP showing particularly strong associations (e.g., TN vs. aboveground fresh weight (AFW), r = 0.724, *p* = 0.012).

**Figure 8:**
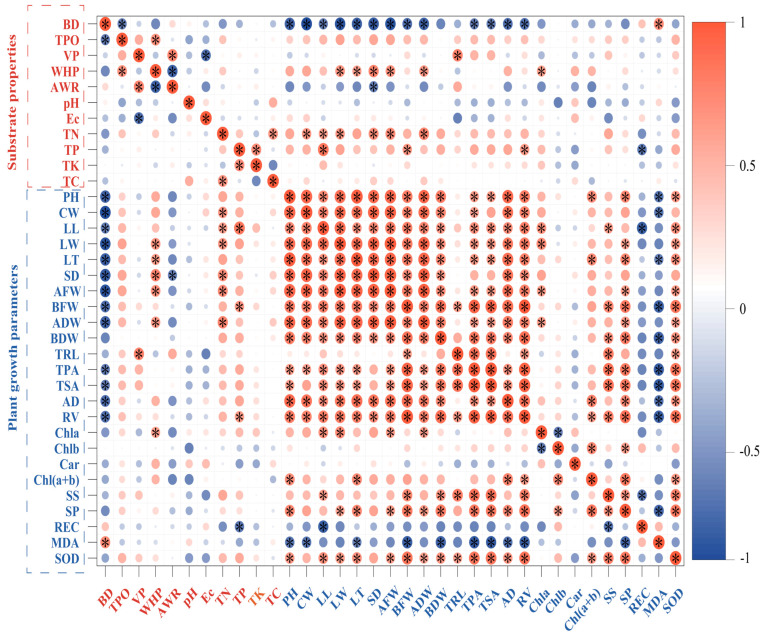
Pearson correlation heatmap between substrate properties and plant parameters. The circle size and color represent the correlation coefficient (R^2^), with red and blue indicating positive and negative correlations, respectively. Asterisks (*) indicate significance at *p* ≤ 0.05.

Several growth traits also exhibited significant positive correlations with soluble sugars (SS), soluble proteins (SP), and superoxide dismutase (SOD), and negative correlations with malondialdehyde (MDA) and relative electrical conductivity (REC) (e.g., SP vs. plant height (PH), r = 0.746, *p* = 0.008; MDA vs. plant height (PH), r = −0.662, *p* = 0.027). In addition, total chlorophyll [Chl(a+b)] was positively correlated with plant growth traits (e.g., Chl(a+b) vs. belowground fresh weight (BFW), r = 0.649, *p* = 0.031) (Figure 8, Table S3).

### 2.8. Comprehensive Evaluation of Media Suitability for Phalaenopsis ‘Big Chili’ Cultivation

The biochar–coconut shell mixtures affected the morphological, physiological, and pigment traits of *Phalaenopsis* ‘Big Chili’, depending on the feedstock type and mixing ratio (Table 1). Membership function analysis identified BC1 (0.876) as having the best performance, followed by BC2 (0.839) and BW2 (0.734), all exceeding the traditional substrate CK (0.683). In contrast, treatments with excessive biochar (e.g., BC3, BZ3, and BW3 with values < 0.4) generally inhibited plant performance. Additionally, BZ treatment (≤0.446) consistently ranked among the lowest.

**Figure 9:**
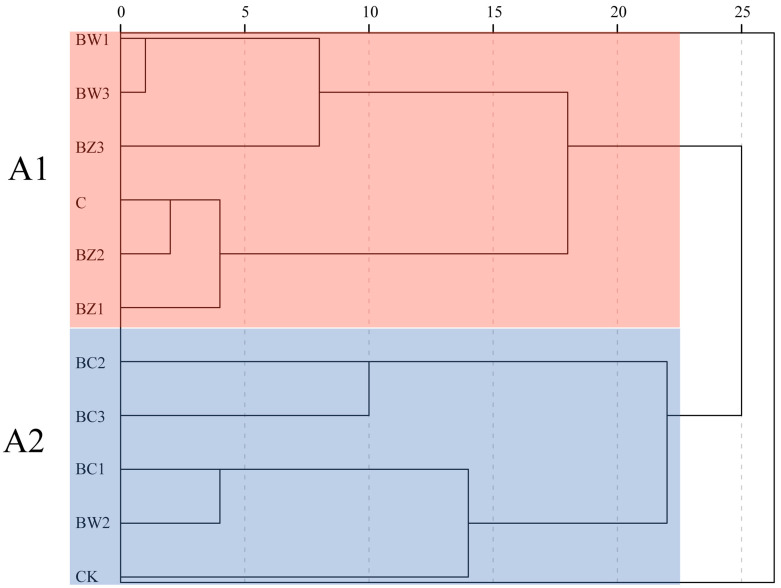
Cluster analysis of substrate treatments based on the measured physicochemical properties. Two distinct clusters were identified: A1 (C, BZ1, BZ2, BZ3, BW1,BW3) and A2 (CK, BC1, BC2, BC3, BW2).

Cluster analysis based on substrate physicochemical properties grouped the treatments into two clusters: A1 (C, BZ1, BZ2, BZ3, BW1, and BW3) and A2 (CK, BC1, BC2, BC3, and BW2) (Figure 9). Cluster A2, particularly BC1, was associated with higher values in plant height, crown width, and leaf size, while Cluster A1 showed overall weaker performance. Compared with Cluster A2, substrates in Cluster A1 exhibited higher BD (0.210 vs. 0.145 g·cm^−3^), lower TPO (51.99% vs. 60.22%) and WHP (18.94% vs. 29.64%), as well as lower TN (2.47 vs. 3.58 g·kg^−1^), TP (0.32 vs. 0.53 g·kg^1^), and TC (44.84% vs. 48.57%) contents. These physicochemical differences may be associated with the observed variation in *Phalaenopsis* ‘Big Chili’ growth performance between the two clusters (Table S4).

## 3. Discussion

### 3.1. Influence of Substrate Physicochemical Properties on Morphological Traits of Phalaenopsis

Significant differences in growth performance among treatments indicate that the physicochemical properties of the substrate play a central role in regulating the growth and development of *Phalaenopsis *‘Big Chili’. These properties influence plant responses by jointly modulating the water-air balance and nutrient availability [25]. Substrates characterized by low bulk density, high total porosity, moderate aeration, and good water-holding capacity (A2) supported efficient water transport, maintained leaf turgor, and enhanced photosynthesis. In contrast, A1 substrates with limited water retention induced mild water stress and slower leaf expansion. Excess moisture in poorly aerated substrates also inhibits growth [26]. Our findings suggest that optimal aboveground development requires a balance between water retention and aeration—consistent with studies showing that low BD, high porosity, and water-holding capacity, along with at least 10% aeration porosity, promote healthy growth—especially in species with fleshy leaves and high transpiration demand, such as *Phalaenopsis* [27,28]. Chemical properties also play a key role [29]. The best-performing treatment, BC1, featured high nitrogen, high potassium, and moderate phosphorus—suggesting that balanced nutrient availability supports the development of aboveground traits. Overall, substrates that combine adequate nutrient supply, sufficient water retention, and moderate porosity provide optimal conditions for leaf development [30].

Consistent with previous findings, our results demonstrated that root development was enhanced in substrates with improved aeration and drainage, highlighting the sensitivity of *Phalaenopsis* roots to substrate structure [31]. In our study, the root index of the control group (CK) was significantly lower than those of BC1, BC2, and BW2, suggesting that biochar-treated substrates—with enhanced water-holding porosity—were more conducive to root development. In contrast, CK exhibited a reduction in root biomass during the later growth stages, likely due to excessive porosity and water retention, which impaired drainage and oxygen availability, ultimately inhibiting root elongation and expansion [32].

In addition to structural traits, chemical characteristics such as pH, electrical conductivity (EC), and nutrient content also influence root development [33]. Although A2 substrates generally supported better shoot growth than A1, root performance in BC3 was notably poor. The biochar used in the BC and BZ treatments was alkaline; BC1, BC2, BZ1, and BZ2 were weakly alkaline (pH < 8.0), while BC3 and BZ3 were strongly alkaline (pH > 8.89). Both BC3 and BZ3 also exhibited high EC values (>1.521 mS·cm^−1^). Elevated pH may reduce micronutrient availability and alter rhizosphere microbial communities, thereby suppressing root development [34]. While *Phalaenopsis* can tolerate moderate pH fluctuations, values beyond their tolerance range impair their performance. Additionally, excess soluble salts may disrupt the shoot–root nutrient balance and weaken the root system over time [35].

The incorporation of biochar–coconut shell mixtures at specific ratios helped achieve an optimal air–water balance, promoting root proliferation. Correlation analysis further supported the association between root growth parameters and physicochemical substrate traits. As root systems expand, high bulk density restricts growth, whereas biochar mixtures reduce bulk density and increase porosity. A 1:10 biochar-to-coconut shell ratio resulted in the highest total porosity, facilitating fleshy root growth under aerobic conditions.

### 3.2. Effects on Physiological Responses and Integrated Performance of Phalaenopsis

Physiological indices, such as chlorophyll content and stress-responsive metabolites, are important indicators for evaluating photosynthetic performance and stress adaptation in plants [36,37]. In this study, the combination of corn straw biochar and coconut shell, particularly in the BC1 and BC2 treatments, led to increased levels of soluble sugars (SS) and soluble proteins (SP) and a reduction in malondialdehyde (MDA) content. These results suggest that biochar application enhances metabolic activity and alleviates oxidative stress, likely through improved substrate structure and rhizosphere conditions that facilitate water and nutrient uptake [38].

While chlorophyll a and carotenoid concentrations were relatively higher in BC1 and BC2, there were no significant differences in total chlorophyll content (a + b) across treatments. This suggests that although suitable biochar addition may indirectly support pigment biosynthesis via improved moisture and nutrient availability, its impact on total chlorophyll accumulation in Phalaenopsis is limited. This finding is consistent with previous research, indicating that chlorophyll synthesis in this species is only marginally affected by substrate composition [39].

Correlation analysis showed that photosynthetic pigment levels were positively associated with total porosity, water-holding capacity, and air–water ratio, whereas SS and SP levels were negatively correlated with high pH and EC values. These results indicate that excessive alkalinity or salinity may induce physiological stress, reinforcing the notion that both the physical and chemical properties of the substrate jointly regulate plant physiological responses [40].

The patterns observed in the physiological parameters were consistent with the comprehensive evaluation results, in which BC1, BC2, and BW2 achieved the top rankings in morphological, physiological, and pigment-related traits. Rather than reflecting a single dominant factor, these findings point to the need for a balanced substrate formulation that supports coordinated improvements in structure, nutrient availability, and plant metabolism [41].

## 4. Materials and Methods

### 4.1. Materials and Growing Medium Preparation

Three biomass feedstocks—corn (*Zea mays*), bamboo (*Bambusa* spp.), and walnut (*Juglans regia*)—were used to produce biochar. The raw materials (corn stalks, bamboo stems, and walnut shells) were collected from agricultural and forestry areas in Zhengzhou, Henan Province, China. After simple impurity removal, the materials were placed in a laboratory pyrolysis furnace and carbonized under oxygen-limited conditions. Pyrolysis was conducted at 500 °C, 700 °C, and 600 °C for corn straw, bamboo, and walnut shells, respectively, with a duration of 2 h. After pyrolysis, the materials were cooled to room temperature and air-dried for one week [42]. The coconut shell, commercially sourced from a supplier in Jinhua (Zhejiang Province, China), was soaked in water for 3 days to ensure full expansion. The water was renewed until the electrical conductivity (EC) dropped below 0.5 mS/cm. The shells were then dehydrated using an industrial dewatering machine (Model: SS755-500) in 30-s cycles until only slight moisture was released upon gentle finger pressure. Similarly, sphagnum moss imported from Chile was soaked for 4 h to remove residual impurities and then processed using the same dewatering method [43].

### 4.2. Experimental Design

The study was conducted from June to November 2024 in a climate-controlled greenhouse at Xinzhenyu Biotechnology Co., Ltd. (Zhangzhou, China). Acclimatized tissue-cultured *Phalaenopsis *‘Big Chili’ seedlings from a single batch were transplanted into 4.3 cm transparent containers with one seedling per pot. Eleven substrate treatments were included, with 60 pots each. The treatments consisted of a control using pure sphagnum moss (CK), a group with 100% coconut shell (C), and nine combinations of coconut husk mixed with one of three biochar types—maize straw (BC), bamboo (BZ), and walnut shell (BW) biochars mixed with coconut shell at volume ratios of 1:2, 1:10, or 4:1. The treatment groups and their respective substrate ratios are presented in Table 1. Three volume ratios (1:2, 1:10, and 4:1) were selected based on preliminary exploratory trials designed to evaluate the response range of *Phalaenopsis *‘Big Chili’ to varying levels of biochar addition. These ratios represent high (4:1), moderate (1:2), and low (1:10) biochar application levels Table 2). Preliminary observations indicated that these proportions elicited distinct plant responses, making them suitable for assessing the influence of biochar content across a representative range. To ensure consistency and comparability, the same ratios were applied to all biochar types.

### 4.3. Analysis of the Properties of Growing Media

The physical properties of the substrates, including bulk density, total porosity, ventilation porosity, water-holding porosity, and air-to-water ratio, were determined using the core method [44]. Air-dried substrate samples were packed into 100 mL stainless steel rings and submerged in water for 24 h to ensure complete saturation. The rings were then blotted to remove surface moisture, and the saturated weight (W_2_) was recorded. After natural drainage, the drained weight (W_3_) was measured. The samples were subsequently oven-dried at 65 °C to a constant weight (W_4_). The empty weight of the ring (W_0_) and its weight after filling with air-dried substrate (W_1_) were also recorded. The following formulas were used:

Bulk density (BD) (g·cm^−3^) = (W_4_ − W_0_)/100

Total porosity (TPO) (%) = (W_2_ − W_4_)/100 × 100%

Ventilation porosity (VP) (%) = (W_2_ − W_3_)/100 × 100%

Water-holding porosity (WHP) (%) = TPO − VP

Air-to-water ratio (AWR) = VP/WHP

Chemical properties, including pH, electrical conductivity (EC), total carbon (TC), total nitrogen (TN), total phosphorus (TP), and total potassium (TK), were analyzed using standard procedures. Substrate samples were mixed with distilled water at a 1:5 (*v*/*v*) ratio, shaken for 10 min, and allowed to stand for 1 h before pH and EC were measured using calibrated meters [45].

TC was measured using the Walkley–Black dichromate oxidation method. A 0.01 g sample was oxidized with 0.167 M K_2_Cr_2_O_7_ and H_2_SO_4_, heated, diluted, and back-titrated with Fe(NH_4_)_2_SO_4_ using a ferroin indicator [46].

Total nitrogen (TN) was measured using the Kjeldahl method. A 0.50 g sample was digested with H_2_SO_4_ and a catalyst, diluted to 100 mL, and distilled after the addition of NaOH. The released ammonia was absorbed in boric acid and titrated with 0.0215 M HCl (blank = 0.09 mL) [47].

Total phosphorus (TP) was determined colorimetrically at 420 nm using the vanadomolybdate method after H_2_SO_4_–H_2_O_2_ digestion of a 0.50 g sample. A 10 mL aliquot was reacted with the reagent and quantified using a standard curve [48].

Total potassium (TK) was analyzed by flame photometry (λ = 766.5 nm) following digestion of a 0.50 g sample with 10 mL H_2_SO_4_ and 5 mL 30% H_2_O_2_ at 180 °C, then diluted to 100 mL [49].

### 4.4. Measurements of Plant Growth Parameters

Growth measurements were taken at 0, 30, 60, 90, 120, and 150 days after transplantation. Parameters included plant height (PH), leaf length (LL), leaf width (LW), leaf thickness (LT), stem diameter (SD), and crown width (CW). The PH was measured from the substrate surface to the plant’s highest natural point. LL, LW, and LT were measured on the second fully expanded leaf from the top at its longest, widest, and thickest points, respectively. SD was measured 1 cm above the root–shoot junction at the narrowest point of the stem. CW was defined as the maximum horizontal spread of the fully extended leaves.

### 4.5. Measurement of Root Parameters

Roots were gently washed with distilled water, arranged in transparent trays, and counted per plant. The roots were then scanned at 300 dpi using a root scanner (HM-GX02; Meiheng, Weifang, China). Root images were analyzed using WinRHIZO Pro 32-bit (version 2017a, released 12 January 2017; Regent Instruments Inc., Québec City, QC, Canada) to obtain the total root length (TRL), total projected area (TPA), total surface area (TSA), average diameter (AD), and root volume (RV) [50].

### 4.6. Measuring Plant Biomass

At 150 days after transplantation, six plants were randomly selected from each treatment group for biomass assessment [51]. Each plant was separated into shoots and roots. The roots were then rinsed with distilled water and blotted dry. The fresh weights were measured using a precision balance (±0.001 g). Samples were dried at 105 °C for 30 min, then at 65 °C to a constant weight, after which dry weights were recorded.

### 4.7. Measurement of Plant Physiological Parameters

At 150 days after transplantation, the second fully expanded leaf from each plant was collected for physiological analysis.

The relative electrical conductivity (REC) was measured to assess membrane permeability. Leaf tissue (0.1 g) was soaked in 10 mL deionized water for 24 h, and the initial conductivity (S_1_) was measured. The samples were then heated at 100 °C for 15 min, cooled to room temperature, and the final conductivity (S_2_) was measured. The REC was calculated as (S_1_/S_2_) × 100% [52].

The soluble sugar content was determined using a commercial assay kit (Shanghai Liquid–Mass Spectrometry Detection Technology Co., Ltd., Shanghai, China) based on the sulfuric acid–anthrone colorimetric method. A 0.1 g sample was ground with 1 mL distilled water, heated at 100 °C for 10 min, centrifuged at 4000 rpm for 10 min, and the absorbance of the supernatant was measured at 620 nm [53].

For soluble protein (SP), superoxide dismutase (SOD), and malondialdehyde (MDA), five leaves per treatment were homogenized in 1 mL of cold 50 mM phosphate buffer (pH 7.8), followed by 4 mL of additional buffer. The homogenate was centrifuged at 12,000 rpm for 20 min at 4 °C, and the supernatant was used for assays.

SP was measured using the Coomassie Brilliant Blue method [36] by mixing 0.5 mL of the extract with 5 mL of dye reagent and measuring the absorbance at 595 nm after 2 min.

SOD activity was determined using the NBT photoreduction method. The extract (0.1 mL) was mixed with 3 mL of the reaction solution, exposed to light for 20 min (with a dark control), and the absorbance was measured at 560 nm. One unit of SOD activity was defined as the amount of enzyme that inhibited NBT reduction by 50%.

MDA content was determined using the thiobarbituric acid (TBA) method. The extract (1 mL) was mixed with 3 mL TBA reagent, heated at 100 °C for 15 min, cooled, centrifuged at 4000 rpm for 10 min, and the absorbance of the supernatant was recorded at 600, 532, and 450 nm.

### 4.8. Measurement of Photosynthetic Pigments

Photosynthetic pigments were determined using ethanol extraction. Fresh leaves were cut into small pieces and soaked in 95% ethanol in the dark for 48 h. Absorbance was measured at 665, 649, and 470 nm, and the pigment concentrations were calculated [54].

### 4.9. Calculations and Statistical Analysis

A one-way analysis of variance (ANOVA) was used to assess the impact of substrate composition on the physical and chemical properties of the substrates, as well as on the characteristics of the plant. Duncan’s multiple range test was employed to determine statistical differences among the treatments (*p* ≤ 0.05). Pearson’s correlation analysis was conducted to assess the relationships between plant traits and substrate properties.

To comprehensively evaluate the overall performance of *Phalaenopsis* ‘Big Chili’ seedlings, a membership function analysis was conducted based on 25 measured indicators, including morphological, physiological, and biochemical characteristics. Each trait was assigned an equal weight. For traits that were positively associated with plant performance, the membership function value was calculated as follows:

R(X_i_) = (X_i_ − X_min_)/(X_max_ − X_min_) (1)

For negatively associated traits (e.g., REC and MDA), an inverse function was used:

R(X_i_) = 1 − (X_i_ − X_min_)/(X_max_ − X_min_) (2)

The average membership value ($\bar{R}$) for each treatment was calculated as:

(3) $$\bar{R}(X_{i})=\frac{R{(X}_{1})+R(X_{2})+...+R(X_{n})}{n}$$

The average membership value ($\bar{R}$) for each treatment was calculated as the arithmetic mean of all 25 indicator-specific R values. Higher $\bar{R}$ values indicated superior integrated performance across all evaluated plant traits. Treatments were ranked accordingly, with rank 1 denoting the best-performing substrate and rank 11 denoting the worst.

To further explore potential groupings among treatments based on substrate physicochemical properties, hierarchical cluster analysis was performed using Ward’s method, with Euclidean distance as the dissimilarity measure.

All statistical analyses were performed using IBM SPSS Statistics version 27 (IBM Corp., Armonk, NY, USA).

## 5. Conclusions

In this study, the incorporation of three types of biochar (corn straw, bamboo, and walnut) with coconut shell in specific proportions significantly improved the growth, physiological performance, and pigment content of *Phalaenopsis *‘Big Chili’. Through the interaction of their physical and chemical properties, the biochar-coconut shell mixtures formed a growing substrate characterized by low bulk density, high total porosity, moderate ventilation porosity, high water-holding porosity, and favorable chemical composition. These properties effectively promoted the growth and development of both the aboveground and belowground parts of *Phalaenopsis*. Among all treatments, BC1 (maize straw biochar: coconut shell = 1:2) achieved the highest comprehensive score, and BC2 and BW2 also outperformed the traditional control. Overall, incorporating biochar into coconut shell substrates at appropriate ratios enhances the growth of *Phalaenopsis ‘Big Chili’*. We recommend BC1 as an effective substrate component for commercial cultivation.

## Figures and Tables

**Table 1:** Comprehensive evaluation of the comprehensive traits of *Phalaenopsis* ‘Big Chili’ under different treatments.

Trait	CK	C	BC1	BC2	BC3	BZ1	BZ2	BZ3	BW1	BW2	BW3
PH	0.736	0.188	1.000	0.864	0.538	0.141	0.070	0.000	0.658	0.725	0.392
CW	0.725	0.174	1.000	0.848	0.671	0.378	0.333	0.000	0.501	0.563	0.391
LL	0.859	0.000	1.000	0.580	0.156	0.491	0.278	0.081	0.223	0.802	0.137
LW	0.968	0.152	1.000	0.863	0.559	0.250	0.289	0.000	0.306	0.867	0.196
LT	0.897	0.437	0.960	1.000	0.643	0.162	0.119	0.000	0.683	0.885	0.481
SD	0.993	0.293	0.998	1.000	0.702	0.179	0.162	0.000	0.378	0.544	0.212
AFW	0.956	0.322	1.000	0.984	0.643	0.302	0.227	0.000	0.376	0.823	0.379
BFW	0.617	0.428	1.000	0.791	0.204	0.308	0.483	0.000	0.498	0.756	0.431
ADW	0.712	0.116	0.794	1.000	0.496	0.107	0.078	0.000	0.236	0.667	0.223
BDW	0.150	0.006	1.000	0.984	0.110	0.065	0.264	0.000	0.503	0.680	0.194
TRL	0.436	0.561	0.606	0.929	0.000	0.692	1.000	0.118	0.651	0.882	0.400
TPA	0.629	0.506	0.963	1.000	0.079	0.437	0.768	0.000	0.649	0.960	0.464
TSA	0.638	0.514	0.963	1.000	0.079	0.437	0.768	0.000	0.649	0.972	0.464
AD	0.660	0.332	1.000	0.587	0.378	0.074	0.227	0.000	0.437	0.545	0.494
RV	0.606	0.384	1.000	0.821	0.142	0.247	0.484	0.000	0.511	0.763	0.409
Chla	1.000	0.000	0.866	0.539	0.872	0.427	0.463	0.595	0.178	0.934	0.378
Chlb	0.382	0.786	0.392	0.609	0.031	0.000	0.278	0.131	1.000	0.184	0.682
Car	0.426	0.026	0.051	0.350	1.000	0.000	0.537	0.281	0.137	0.138	0.027
Chl(a + b)	0.941	0.614	0.847	0.735	0.438	0.000	0.398	0.333	1.000	0.661	0.794
SS	0.672	0.516	0.891	1.000	0.216	0.000	0.594	0.187	0.926	0.767	0.452
SP	0.645	0.368	0.706	1.000	0.000	0.584	0.628	0.286	0.550	0.645	0.532
REC	0.304	1.000	1.000	0.916	0.350	0.922	0.874	0.524	0.373	0.920	0.376
MDA	0.458	0.782	0.979	0.769	0.530	0.305	0.900	0.000	0.889	1.000	0.699
SOD	0.976	0.750	1.000	0.976	0.000	0.441	0.471	0.275	0.823	0.939	0.396
$\bar{R}$(Xᵢ)	0.683	0.386	0.876	0.839	0.368	0.290	0.446	0.117	0.547	0.734	0.400
Rank	4	8	1	2	9	10	6	11	5	3	7

**Table 2:** Different ratio treatment groups.

Treatment	Substrates
CK	Sphagnum moss
C	Coconut shell
BC1	Maize straw biochar:Coconut shell = 1:2
BC2	Maize straw biochar:Coconut shell = 1:10
BC3	Maize straw biochar:Coconut shell = 4:1
BZ1	Bamboo biochar:Coconut shell = 1:2
BZ2	Bamboo biochar:Coconut shell = 1:10
BZ3	Bamboo biochar:Coconut shell = 4:1
BW1	Walnut shell biochar:Coconut shell = 1:2
BW2	Walnut shell biochar:Coconut shell = 1:10
BW3	Walnut shell biochar:Coconut shell = 4:1

## Data Availability

All data generated or analyzed during this study are included in this published article and are also available from the corresponding author upon reasonable request.

## Supplementary Materials

The following supporting information can be downloaded at: https://www.mdpi.com/article/10.3390/plants14142092/s1, Table S1: ANOVA results for the physical properties of the treatment groups. Table S2: ANOVA results for the chemical properties of the treatment groups. Table S3: Pearson correlations among substrate properties and growth-related traits of *Phalaenopsis* ‘Big Chili’. Table S4: Mean values of substrate physicochemical properties for the two clusters identified in the hierarchical cluster analysis. Figure S1: Time-course changes in aboveground growth traits of Phalaenopsis under different substrate treatments from 30 to 120 days.
