# Supplementary material for: Biochar-Coconut Shell Mixtures as Substrates for Phalaenopsis ‘Big Chili’
Source: Plants (Basel). 2025 Jul 8;14(14):2092. doi: 10.3390/plants14142092 (PMC12297926; doi:10.3390/plants14142092)
Supplement: Supplementary file 1 [file plants-14-02092-s001.zip › Supplementary File/Supplementary File.docx]

**Table S1.** ANOVA results for the physical properties of treatment groups.

| **Treatment** | **BD(g/cm^-3^)** | **TPO(%)** | **VP(%)** | **WHP(%)** | **AWR(%)** |
| --- | --- | --- | --- | --- | --- |
| CK | 0.145±0.002g | 74.76±0.92a | 31.95±1.59cd | 42.81±1.16a | 0.75±0.05f |
| C | 0.216±0.003b | 59.97±0.70b | 40.15±1.50ab | 19.82±0.81ef | 2.04±0.16bc |
| BC1 | 0.143±0.002g | 52.58±1.27cd | 28.28±1.70de | 24.30±0.90d | 1.17±0.10e |
| BC2 | 0.145±0.002g | 61.07±0.87b | 32.81±0.98c | 28.26±0.76c | 1.16±0.06e |
| BC3 | 0.139±0.001g | 54.26±1.32c | 23.11±1.05f | 31.15±1.50b | 0.75±0.06f |
| BZ1 | 0.202±0.004c | 53.00±1.15c | 37.94±0.72ab | 15.05±1.33g | 2.57±0.26a |
| BZ2 | 0.167±0.001e | 61.00±1.28b | 41.78±1.48a | 19.22±0.95ef | 2.19±0.17ab |
| BZ3 | 0.288±0.005a | 41.41±0.76f | 23.12±1.23f | 18.29±0.49f | 1.27±0.10e |
| BW1 | 0.175±0.001d | 49.81±0.74d | 29.56±1.61cd | 20.25±0.89ef | 1.47±0.15de |
| BW2 | 0.153±0.002f | 58.43±0.12b | 36.75±0.92b | 21.68±0.83de | 1.70±0.11cd |
| BW3 | 0.215±0.002b | 46.76±1.14e | 25.07±0.62ef | 21.02±0.786ef | 1.20±0.06e |
| F_10,22_ | 310.585 | 79.834 | 27.608 | 62.395 | 19.828 |
| P | < 0.001 | < 0.001 | < 0.001 | < 0.001 | < 0.001 |

Note: Values are means ± standard error (n = 3). BD = bulk density; TPO = total porosity; VP = ventilation porosity; WHP = water-holding porosity; AWR = air–water ratio. Different letters indicate *P* ≤0.05 significance (Duncan’s test).

**Table S2.** ANOVA results for the chemical properties of the treatment groups.

| **Treatment** | **pH** | **EC(ms·cm^-1^)** | **TN(g·kg^-1^)** | **TP(g·kg^-1^)** | **TK(g·kg^-1^)** | **TC(%)** |
| --- | --- | --- | --- | --- | --- | --- |
| CK | 4.80±0.01j | 0.143±0.005h | 3.49±0.06c | 0.459±0.008d | 5.76±0.02c | 41.77±0.86e |
| C | 6.21±0.03g | 0.111±0.006i | 2.58±0.11e | 0.276±0.010ef | 5.37±0.04d | 50.38±1.13c |
| BC1 | 7.99±0.03c | 1.147±0.006d | 3.57±0.02c | 0.980±0.008a | 7.22±0.06b | 46.34±0.62d |
| BC2 | 7.76±0.04d | 0.230±0.005f | 5.00±0.03a | 0.292±0.007e | 2.96±0.02g | 57.57±0.65a |
| BC3 | 8.89±0.03b | 1.521±0.002b | 2.84±0.02d | 0.171±0.009h | 4.54±0.01f | 53.96±0.89b |
| BZ1 | 7.47±0.03e | 1.446±0.007c | 3.75±0.05b | 0.604±0.016c | 5.61±0.08cd | 50.42±1.35c |
| BZ2 | 7.07±0.02f | 0.244±0.005f | 1.93±0.02g | 0.255±0.012fg | 5.39±0.04d | 42.45±0.61e |
| BZ3 | 9.00±0.01a | 2.885±0.003a | 2.10±0.04f | 0.234±0.005g | 5.53±0.09cd | 42.95±0.95e |
| BW1 | 5.38±0.02h | 0.175±0.004g | 2.17±0.08f | 0.280±0.010ef | 4.87±0.01e | 41.47±0.44e |
| BW2 | 6.20±0.02g | 0.085±0.005j | 3.01±0.09d | 0.722±0.014b | 8.33±0.21a | 43.22±0.77e |
| BW3 | 5.26±0.03i | 0.305±0.006e | 2.26±0.04f | 0.259±0.008fg | 5.73±0.06c | 41.37±0.37e |
| F_10,22_ | 3122.443 | 31170.907 | 253.858 | 608.869 | 295.172 | 46.349 |
| P | < 0.001 | < 0.001 | < 0.001 | < 0.001 | < 0.001 | < 0.001 |

Note: Values are means ± standard error (n = 3). EC = electrical conductivity; TN = total nitrogen; TP = total phosphorus; TK = total potassium; TC = total carbon. Different letters indicate *P* ≤0.05 significance (Duncan’s test).


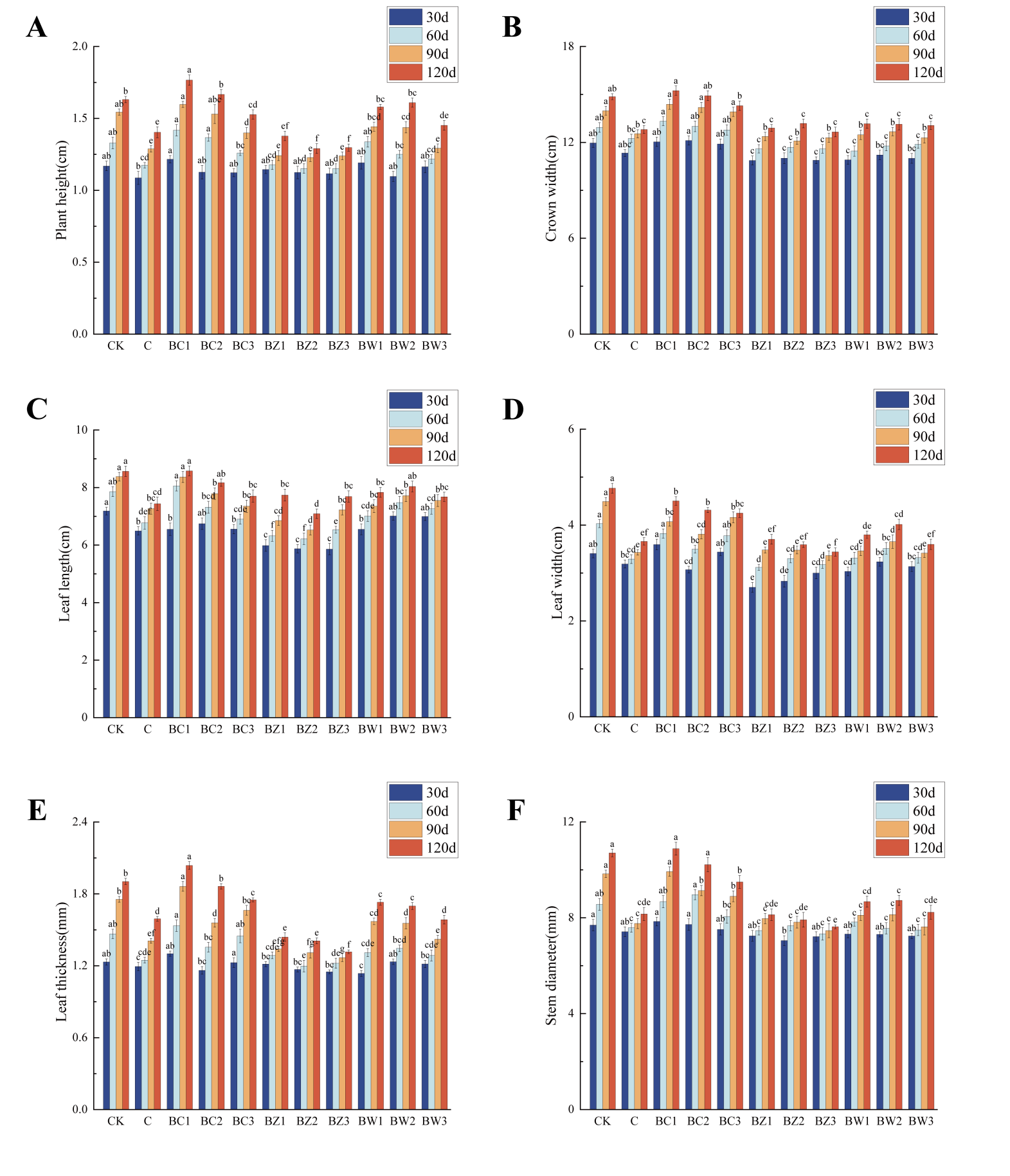


**Figure S1**. Time-course changes in aboveground growth traits of Phalaenopsis under different substrate treatments from 30 to 120 days. (A) Plant height (PH), (B) Crown width (CW), (C) Leaf length (LL), (D) Leaf width (LW), (E) Leaf thickness (LT), (F) Stem diameter (SD). Measurements were taken at 30, 60, 90, and 120 days after transplanting. Values are means ± SE (n = 15). Different letters indicate significant differences among treatments at the same time point *P* ≤0.05, (Duncan’s test).

**Table S4**. Mean values of substrate physicochemical properties for the two clusters identified in the hierarchical cluster analysis.

| **Variable** | **Cluster A1 Mean** | **Cluster A2 Mean** |
| --- | --- | --- |
| BD(g/cm^-3^) | 0.210 | 0.145 |
| TPO(%) | 51.992 | 60.221 |
| VP(%) | 32.938 | 30.580 |
| WHP(%) | 18.942 | 29.641 |
| AWR(%) | 1.789 | 1.107 |
| pH | 6.732 | 7.211 |
| Ec(ms·cm^-1^) | 0.861 | 0.625 |
| TN(g·kg^-1^) | 2.466 | 3.584 |
| TP(g·kg^-1^) | 0.318 | 0.525 |
| TK(g·kg^-1^) | 5.415 | 5.761 |
| TC(%) | 44.840 | 48.572 |

Note: Clustering was based on standardized values using Ward’s method with Euclidean distance. Mean values are based on original (unstandardized) data.
