# Supplementary figures and images for: Biochar-Coconut Shell Mixtures as Substrates for Phalaenopsis ‘Big Chili’
Source: Plants (Basel). 2025 Jul 8;14(14):2092. doi: 10.3390/plants14142092 (PMC12297926; doi:10.3390/plants14142092)

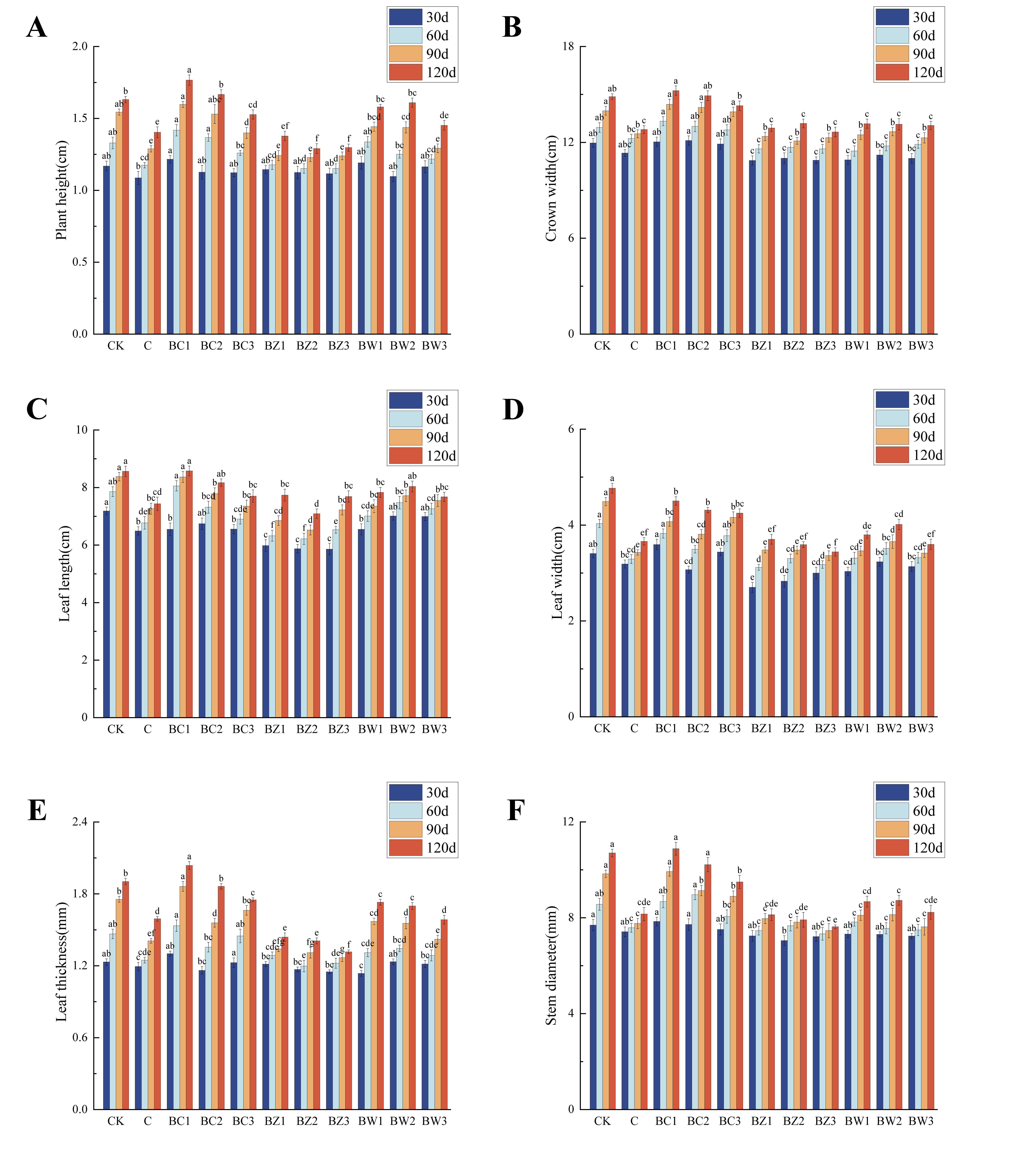

Supplement: Supplementary file 1 [file plants-14-02092-s001.zip › Supplementary File/Figure S1.tif]
